# Hermaphrodite [?] in Insane Asylum

**Published:** 1890-12

**Authors:** F. S. White

**Affiliations:** Ass’t Supt. S. L. A., Terrell


For Daniel's Texas Medical Journal.

### A HERMAPHRODITE (?) IN INSANE ASYLUM.

BY F. S. WHITE, M. D., ASS'T SUPT. S. L. A., TERRELL.

Read at Terrell Medical Association meeting, and voted to Daniel's Texas Medical Journal.

CHRISTINA HOFFMAN, married, aged 38, insane for six years. Had been confined in the North Texas insane asylum for five years. Was born in Germany. Husband a Bohemian. Were married in Illinois, and had been living as man and wife for several years.

This patient had been at one time confined in an insane asylum in Illinois, but was discharged. Was of a very marked masculine appearance, and had a considerable growth of beard; voice decidedly masculine, as was the entire make up and appearance. When in health would have weighed about 140. About one year prior to death it was discovered that there was some malformation about the genitals, but the exact nature was not determined, and it was supposed that this person was essentially a woman, she having always dressed in female attire, and associated with women; she also being a man's wife.

Death was caused by pulmonary tuberculosis.

Autopsy eight hours after death. Body very much emaciated, bones all very large and joints prominent; no mammary glands; chest large and masculine. The pubic bones were rather prominent.

General appearance that of a man. There was no trace of a uterus or ovaries. There were two small testicles, which measured about one inch by three-fourths. The right was found just above, and lying alongside of Poupart's ligament. The left was found occupying the labia majora, just a little below the attachment of Poupart's ligament to the pubic bone. The labia majora were tolerably well developed, but not quite so round and prominent as in the well formed female.

There was only a rudiment of a vagina, only a shallow fold of mucous membrane between the labia majora, and one-half inch in depth, large enough to cover the first finger. The meatus urinarius was in about the normal location, and beneath it was a very small transverse band of mucous membrane. The clitoris was very much enlarged and elongated, in fact had more the appearance of a penis; was about two inches in length, the labia minora forming a first-class foreskin for it, and there was a tolerably well developed glans, but no urethra. This organ was decidedly erectile, as I ascertained after death, and was capable of becoming very hard, and putting on very much the appearance of a ten year-old boy's penis.

Under the foreskin was an enormous accumulation of smegma, showing that the glands which are peculiar to the penis were present and performing their normal function. On one or two occasions this creature had made amorous advances toward one of the female patients and also toward one of the female attendants, but was not troublesome in the least; but these demonstrations were sufficient to show that there existed some of the animal passion.

I suppose that during intercourse with her [?] husband sufficient friction was produced in this clitoris or penis to complete the orgasm.

